# The challenges of lockdown for early-career researchers

**DOI:** 10.7554/eLife.59634

**Published:** 2020-06-12

**Authors:** Nicola Byrom

**Affiliations:** Institute of Psychiatry, Psychology and Neurosciences, King’s College LondonLondonUnited Kingdom

**Keywords:** mental health, academia, covid-19, COVID-19 and the Research Community

## Abstract

Thousands of UK doctoral students and early-career researchers shared the repercussions of lockdown on their work and wellbeing.

In March 2020, as the COVID-19 pandemic hit the UK, universities closed their doors with no information about when they would reopen. While many undergraduate students made their way home and tried to settle into online learning, most researchers were asked to carry on with their work from home.

Within higher education, both doctoral students and early-career researchers occupy precarious positions. Doctoral researchers – who account for less than 5% of the UK student population – do not identify with the wider student body. Their day-to-day experience is often indistinguishable from salaried researchers, yet they are not employees. As one doctoral student once put it: "There are times when I feel as if I am living in the uncomfortable skin of someone who is seeking validation for the right to grace the halls of academia" (Mason, 2009). Early-career researchers also face many uncertainties: usually working on short-term contracts, worries about the next stage of their career are never far away. In this difficult context, how are these populations faring in their new work environment?

A few weeks after the lockdown announcement, SMaRteN and Vitae launched a survey to explore the impact of COVID-19 on the research community. SMaRtEN, which is based at King’s College London and funded by UK Research and Innovation (UKRI), is a network that supports and encourages better research into student mental health. Vitae is a non-profit programme that works with institutions to enable the professional development of researchers.

The survey was open from April 16 to May 17, with the majority of respondents completing it within the first two weeks. Data were collected online and recruitment facilitated via social media; there will therefore be biases in terms of who responded that could limit the generalisability of the findings.

Over 5900 doctoral researchers and research staff took part, representing approximately 3% of the population of UK doctoral and early-career researchers. The data have been made available for researchers to analyse (www.smarten.org.uk/covid-19-study). Preliminary analyses from the first 4800 respondents are presented below, with over three-quarters currently completing a PhD (Figure 1).

**Figure 1.**
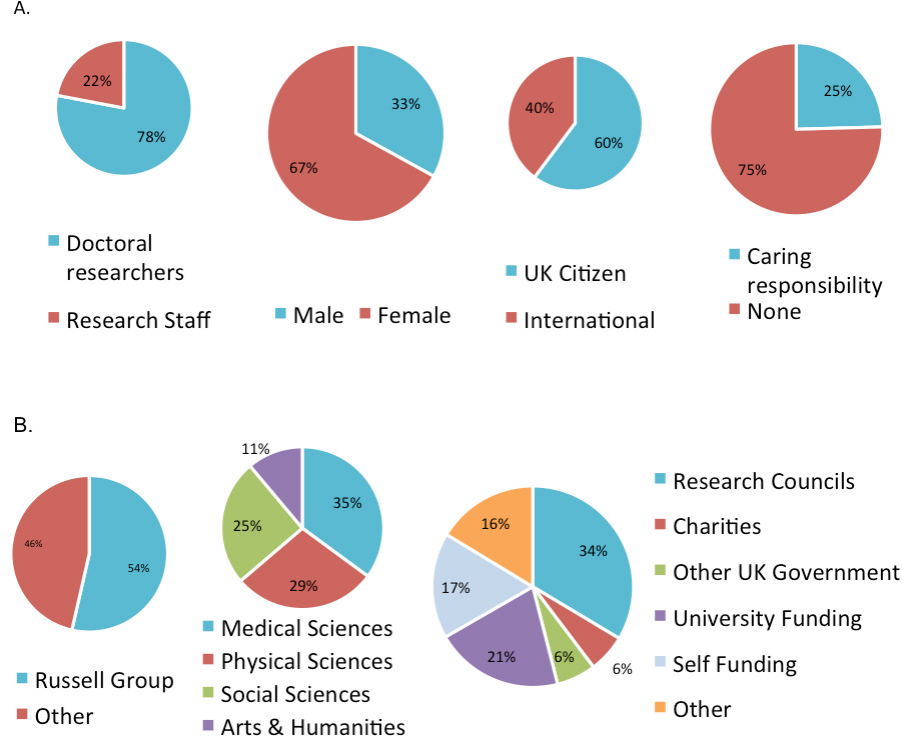
Profile of early respondents. (**A**) Demographic information of the respondents. (**B**) Type of university, field of study and funding origin of the respondents.

While this survey can give us a snapshot into how this population is doing in the context of COVID-19, it is important to note that the quantitative data cannot tell us anything about causation. In particular, we do not know whether observations of poor mental health among this population are specific to COVID-19.

The pandemic and the resulting lockdown have highlighted the precarious employment position of early-career researchers.

## Impaired research

We found that more than three-quarters of respondents were experiencing a negative impact of the lockdown on their ability to collect data, discuss ideas and findings with colleagues, and disseminate research findings. More than half also identified a negative impact on data analysis, writing, and working on grant or fellowship applications. In addition, almost a third of respondents identified that they had reduced or no access to the software that they needed for research, highlighting the importance for universities to provide better access to essential work resources. This decreased ability to work is creating stress: half of respondents reported being very stressed about their work, and two-thirds are very worried about their future plans.

Concern about the future is real: only 12% of final-year doctoral students reported that their institution had provided an option to extend their studies. While UKRI has made some provisions for additional funding, it supports only a minority of doctoral students. There is a general lack of certainty among this population about whether they will be allowed to extend their studies, and whether funding will be available to support them to do so. Among research staff, 40% reported that their research contract ends during 2020, and only 10% of this group report that their funding has been extended in the context of the pandemic. The pandemic and the resulting lockdown have highlighted the precarious employment position of many early-career researchers, and should encourage the sector to consider whether it is morally acceptable to continue the practice of short-term employment contracts.

## Poor mental wellbeing

The survey also collected data on mental wellbeing, distress and loneliness. Three-quarters of respondents showed low levels of mental wellbeing and four in five showed some level of mental distress. Experiences are not equal, however: individuals working in arts and humanities reported higher levels of mental distress and loneliness. Researchers with a physical disability or long-term illness can also find it challenging to work from home if they do not have access to the assistive technology they require. Finally, international researchers working in the UK are lonelier and more stressed about many aspects of day-to-day life, living arrangements, finances and work. Overall, our data suggest that researchers who are less likely to have strong social support networks within and beyond academia in the UK may be more likely to be struggling with their mental wellbeing. Universities should be exploring how they can facilitate work-related social support online. More complex analyses are now required to also tease out the potential effect of gender and caregiving responsibilities on wellbeing.

## Improving the experience of researchers

The survey also explored the resources and skills needed to work effectively from home. While around 60% of respondents identified that their university had made arrangements for skills training to take place online, more attention here may be beneficial, as our analysis indicates that access to online skills training is connected to better mental wellbeing.

We also found links between levels of mental distress and support provided by supervisors or line managers. Two-thirds of respondents reported that they felt their supervisor or line manager had done all they could or should do to provide support at this time. Where strong support was identified, respondents also reported lower levels of mental distress. It is vital to remember that the supervisors and line managers have also been impacted by the lockdown. This is something that many respondents recognised and commented on — understanding, for example, that there may have been certain gaps in support because supervisors currently have children at home. However, continued strong support from supervisors will help early-career researchers.

Around half of respondents identified that their supervisor or line manager had made arrangements to support them to stay in touch with peers and colleagues at their university; these respondents also reported lower levels of loneliness. Online lab groups and virtual catch-ups really can work. Most researchers may be physically distant at present, but there are fantastic opportunities for digital connection, including with colleagues working around the world. At the start of the lockdown SMaRteN set up a virtual, weekly lab group for early-career researchers and doctoral students working on student mental health (www.smarten.org.uk/lab-group). This is just like any other lab group – we have guest speakers and we discuss papers. The only exception is that we have been able to bring together researchers from all across the UK and recently, from Australia.

Universities should be prioritising their doctoral and early-career researchers, a population that is already in a precarious employment position, and whose members now face serious uncertainties around their future within academia. Many have seen months or even years of work disrupted or destroyed by the rapid closure of laboratories; others have had fieldwork cancelled and face-to-face training opportunities curtailed. Those with children or other caring responsibilities are struggling with the reality that they cannot work, while peers around them seem to be ‘getting ahead’. Universities and funders must take on some responsibility for helping this community cope with the disruptions to their work.
